# Global Trends and Research Hotspots of Interventions for Post-Stroke Cognitive Impairment: A Bibliometric Analysis from 2005 to 2025

**DOI:** 10.3390/healthcare14193210

**Published:** 2026-09-28

**Authors:** Shaofei Li, Wei Sun, Benke Zhao, Aini He, Xuefan Yao, Houlin Lai, Kehui Ma, Jiaxin Gao, Jiayin Xiao, Luda Feng, Haiqing Song

**Affiliations:** 1 Department of Neurology, Xuanwu Hospital, Capital Medical University, Beijing 100053, China; lishf2020@163.com (S.L.); sunwei980615@163.com (W.S.);; 2 Stroke Prevention and Translational Research Center, Xuanwu Hospital, Capital Medical University, Beijing 100053, China; 3 National Stroke Administration Office, Xuanwu Hospital, Capital Medical University, Beijing 100053, China

**Keywords:** post-stroke cognitive impairment, clinical intervention, bibliometric analysis

## Abstract

**Background**: Despite the growing clinical importance of post-stroke cognitive impairment (PSCI), standardized and effective interventions remain limited, and no comprehensive bibliometric analysis has systematically mapped global research trends in PSCI interventions. This study aimed to outline the global research landscape and identify emerging trends and future research directions. **Methods**: Publications regarding clinical interventions for PSCI were retrieved from the Web of Science Core Collection database from 1 January 2005 to 31 December 2025. Utilizing CiteSpace and VOSviewer software, this study conducted a comprehensive bibliometric analysis and visualization of the literature. The analysis encompassed research directions, publication trends, countries/regions, institutions, authors, source journals, and keyword characteristics. **Results**: After removing duplicates and applying strict inclusion criteria, 789 English-language articles and reviews were included. The overall findings indicated that annual publications on PSCI-related clinical interventions steadily increased, with rapid growth since 2015, reaching a peak of 130 publications in 2025. China produced the largest volume of publications (n = 358), with the USA acting as the core hub of international collaborative research. Chinese institutions dominated publication volume, but international institutions had greater research impact. The author network was large but lacked high-productivity core scholars, and cross-team collaboration remained limited. Highly cited studies mainly addressed cognitive assessment, pathological mechanisms, and clinical guidelines. Keyword analysis revealed that research hotspots have gradually shifted from functional evaluation to neural mechanism-based intervention research, and the standardization of outcome assessment has become a research focus. **Conclusions**: Global publications on PSCI interventions show a clear increasing trend, accelerating after 2015. Nevertheless, high-quality, multicenter randomized controlled trials are still insufficient. Future research should prioritize rigorously designed clinical trials to establish solid clinical evidence and formulate unified evaluation criteria for cognitive outcomes.

## 1. Introduction

Post-stroke cognitive impairment (PSCI) represents one of the most prevalent and significantly disabling complications following stroke [1,2]. PSCI refers to any new-onset cognitive decline occurring within 3–6 months after a clinically evident stroke, with severity ranging from mild cognitive impairment to dementia. Post-stroke dementia (PSD) represents the dementia-range end of this spectrum, whereas vascular cognitive impairment (VCI) is a broader construct encompassing cerebrovascular-related cognitive impairment, with PSCI constituting one of its subtypes [3,4,5,6]. In a systematic review and meta-analysis of 27 studies including 39,412 patients with ischemic stroke, PSCI was associated with a higher hazard of recurrent stroke (pooled adjusted HR = 1.59, 95% CI 1.29–1.94) and all-cause mortality (pooled adjusted HR = 2.07, 95% CI 1.65–2.59), compared with patients without PSCI [7]. These poor clinical outcomes pose a heavy socioeconomic burden for healthcare systems worldwide. Given that cognitive preservation is independently associated with functional recovery and long-term survival in patients with stroke, the effective management of PSCI holds profound clinical significance [7,8,9].

Pharmacological approaches have mainly extrapolated from vascular dementia and VCI research. Cholinesterase inhibitors and memantine have shown at most modest and inconsistent cognitive benefits, with tolerability and adverse-event concerns in some populations; consequently, they have not been uniformly endorsed as high-level recommendations for routine PSCI treatment [3,4,10,11]. Among non-pharmacological interventions, cognitive training and exercise-based programs have been reported as promising for specific cognitive domains and functional outcomes in some trials and meta-analyses, yet the overall certainty is often limited by small sample sizes, short follow-up, and protocol heterogeneity [12,13,14]. Neuromodulation techniques, including repetitive transcranial magnetic stimulation (rTMS) and transcranial direct current stimulation (tDCS), represent an active and growing area with encouraging signals in some studies, but optimal stimulation targets, dosing parameters, durability of effects, and patient selection remain under debate [15,16,17]. Traditional Chinese medicine–related interventions are also frequently reported, but evidence consistency and external validity remain controversial due to variability in study quality and reporting standards [18,19].

Publications related to PSCI have surged in recent years. However, traditional evidence synthesis methods in this field have mainly focused on relatively specific questions, such as risk and prognosis assessment or the evaluation of single intervention categories [7,20]. While such approaches are essential for estimating intervention effects, they are less suited to global questions about PSCI intervention research [21,22,23]. This study employs bibliometric and visualization methods to identify the main research areas, influential publications and emerging intervention directions in PSCI intervention research, thereby providing a strategic reference for future high-quality clinical investigations and evidence system construction.

## 2. Materials and Methods

### 2.1. Data Sources and Search Strategy

A literature search was conducted using the Web of Science Core Collection (WoSCC) database. This database comprises more than 20,000 globally recognized and influential academic journals. It includes cited references in articles and constructs a distinctive citation index based on cited authors, sources, and publication dates [24]. Given its extensive use in the field of medicine, WoSCC was considered a fitting choice for this study. Eligible studies were limited to peer-reviewed original articles and reviews published in English from 2005 to 2025, with the literature search conducted up to 25 March 2026. To be included, studies had to focus on clinical interventions for adult patients with PSCI, irrespective of gender, and have records with complete bibliographic information. The publications retrieved from WoSCC were saved in plain text format. Supplementary Table S1 lists the specific search strategy.

### 2.2. Data Selection and Standardization

All retrieved records underwent a structured screening process to ensure their relevance to the objectives of this study. Titles and abstracts were initially reviewed to identify publications focusing on clinical interventions for PSCI. Screening was performed based only on titles and abstracts. Full-text assessment was not conducted for this bibliometric analysis. During title and abstract screening, records explicitly referring to PSD were retained and analyzed under the PSCI umbrella, whereas VCI-labeled records were retained only if they involved stroke populations and post-stroke cognitive outcomes; non-stroke VCI records were excluded. Clinical interventions were defined as any interventions aimed at treating or delaying PSCI. Publications that were not directly related to the research topic or did not primarily address clinical interventions for PSCI were excluded.

Following eligibility assessment, the included records were subjected to a standardization procedure to ensure consistency and accuracy in bibliographic information. This process involved harmonizing author names, unifying institutional affiliations, and consolidating synonymous keywords. Duplicate records were carefully identified and removed. These measures were implemented to enhance the reliability and validity of the subsequent bibliometric analyses. Prior to bibliometric analysis, data standardization was conducted. Two reviewers independently screened titles and abstracts. Inter-rater agreement was assessed using Cohen’s kappa, which was 0.85, indicating substantial agreement between the two reviewers. All discrepancies were resolved through discussion. When consensus could not be reached, a third senior reviewer adjudicated the final decision.

### 2.3. Bibliometric and Visualization Analysis

Figure 1 illustrates the workflow from data collection through data analysis to interpretation. Analyses were conducted using VOSviewer 1.6.20 and CiteSpace 6.4.R2. Unless otherwise specified, standard or default settings of the software were used. VOSviewer was used for co-authorship and keyword co-occurrence mapping; node size represents occurrences or publication counts and link strength represents co-occurrence or collaboration intensity. CiteSpace was used for co-citation analysis, betweenness centrality computation, clustering, and burst detection. Betweenness centrality was treated as a descriptive bridging indicator rather than a significance test, and no fixed cut-off was applied. Citation bursts were detected using Kleinberg’s burst detection algorithm as implemented in CiteSpace.

In this study, research hotspots were operationally defined as major themes represented by high-frequency keywords and densely connected keyword co-occurrence clusters in the PSCI intervention literature. Specifically, keyword co-occurrence network analysis and clustering were used to identify hotspot themes. Emerging frontiers were characterized by burst detection of keywords and cited references, with items showing the strongest and most recent bursts considered indicative of rapidly increasing attention. The detailed mathematical formulas and normalization algorithms are provided in (Supplementary Method S1).

## 3. Results

### 3.1. Trend Analysis of Annual Publications

Figure 2 presents the annual and cumulative publication counts from 2005 to 2025. Overall, annual publications increased over time and accelerated after 2015. To visualize the non-linear trend, we applied a second-order polynomial curve as a descriptive smoothing tool. This fit was used for visualization only and not to claim exponential growth. From 2005 to 2014, the field was in its early exploratory stage, with low and fluctuating annual publication volumes. The number of annual publications remained below 25 throughout this decade, starting from 0 in 2005 and peaking at 24 in 2013, while cumulative publications totaled fewer than 107 by 2014. A steady, consistent increase in publication volume began after 2015. The annual number of publications rose from 27 in 2015 to 49 in 2019, and growth became more pronounced after 2020. Specifically, annual publications increased to 59 in 2021, 96 in 2022, and reached the highest value of 130 in 2025. The cumulative number of publications reached 789 by the end of 2025.

### 3.2. National and Institutional Analysis

Research on clinical interventions for PSCI involved 68 countries and regions worldwide. Publication counts, citations, and betweenness centrality for the top 10 countries are presented in Table 1. China led in publication output (n = 358), followed by the USA (n = 115), the United Kingdom (n = 49), Italy (n = 47), and Canada (n = 45). China had the highest total citations (6924), whereas the USA (6490) demonstrated greater average impact despite fewer publications. In terms of citation impact normalized by time, the USA showed a higher average annual citations per paper than China. Notably, Australia had the highest average annual citations per paper (15.5), followed by Germany (12.65) and The Netherlands (10.59), indicating strong per paper annual impact despite fewer publications. Betweenness centrality was highest in the USA (0.48), followed by Italy (0.21), China (0.17), Australia (0.16), and Germany (0.12), highlighting their pivotal roles in international collaboration. Collaboration dynamics are illustrated in Figure 3 and Figures S1 and S2 using a chord diagram, international network map, and density heatmap. The China–USA partnership was the strongest, with additional collaborations between China and several European countries. The network exhibited clustered activity centered on North America, East Asia, and Europe, with China and the USA forming the dual-core of the research network.

In summary, global research on PSCI clinical interventions is centered in China and the USA, characterized by broad international participation alongside high concentration in leading nations.

### 3.3. Institutions

Analysis of publication outputs showed that most institutions contributed only a limited number of papers, whereas a few core institutions accounted for the majority of research output. Table 2 presents the top 10 institutions ranked by publication volume, alongside their citation counts and average citations per document. Capital Medical University led the field with 25 publications, followed by Fujian University of Traditional Chinese Medicine and the University of Toronto. Notably, seven of the top 10 institutions are based in China, highlighting the substantial contribution of Chinese academic institutions to overall research output in this field. While Chinese institutions dominate in publication output, institutions from other countries demonstrate greater research impact, as reflected by higher average citations per document and higher average annual citations per paper. Monash University achieved the highest average citations per document (103.3), followed by the University of Toronto (96.4) and the University of Nottingham (77.1). Consistently, Monash University also showed the highest average annual citations per paper (22.05), followed by the University of Toronto (9.91) and the University of Nottingham (9.56). This indicates a higher citation impact of publications from these institutions despite their lower publication counts. Figure S3 shows the institutional collaboration network colored by publication year. It illustrates evolving research dynamics: early collaborations were centered on international institutions such as the University of Toronto and Monash University, while Chinese institutions including Capital Medical University and Fujian University of Traditional Chinese Medicine emerged as key collaborative hubs after 2015, with extensive domestic partnerships and growing cross-border ties. Figure S4 presents the collaboration network of the top 10 institutions by publication count.

### 3.4. Authors

A total of 4206 authors contributed to research on clinical interventions for PSCI. Most authors published only a small number of papers. Only a small proportion of authors were highly productive: 50 published more than four papers, 22 more than five, and 12 six or more. Table 3 lists the top 12 authors by publication count Calabro, Rocco Salvatore and Tao, Jing were the most productive authors, each contributing 10 publications, followed by Chen, Lidian with 9 papers. In terms of average citations per document, Huang, Jia and De Luca, Rosaria achieved the highest values. In terms of average annual citations per paper, Hu, Xiquan (9.52) and Luo, Jing (9.47) ranked highest, followed by De Luca, Rosaria (8.63). Figure 4 and Figure S5 present the author collaboration landscape, revealing distinct and tightly connected research clusters with relatively limited cross-cluster collaboration.

### 3.5. Journals

Table 4 presents the top 10 journals contributing to PSCI clinical intervention research. *Frontiers in Neurology* leads the field with the highest number of publications (48 papers; journal impact factor (IF) = 2.8; Q2). Meanwhile, *Cochrane Database of Systematic Reviews* and *Stroke* showed the strongest citation impact and the highest IFs, and they also had the highest average annual citations per paper. Notably, 6 of the top 10 journals are classified as Journal Citation Reports (JCR) Q1 or Q2. In addition, the listed journals were primarily from Switzerland (n = 3), the USA (n = 3), and the United Kingdom (n = 3), with one from Italy (n = 1).

Figure 5 and Figure S6 reveal that publications on PSCI clinical interventions are concentrated in journals of neurology, rehabilitation medicine, and evidence-based medicine, forming several closely connected collaborative clusters centered on neuroscience journals. Figure S7 further illustrates the citation and co-citation relationships among journals through an overlay analysis generated using CiteSpace. The citing journals are displayed on the right, whereas the cited journals appear on the left. The thickness of the connecting lines represents the strength of the citation relationship.

The citing journals primarily fall within the fields of clinical neurology, neurorehabilitation, and evidence-based clinical medicine. In contrast, the cited journals can be categorized into two major groups: clinical neurology and rehabilitation medicine journals that largely overlap with the source journals, and journals in basic neuroscience, molecular biology, and immunology that provide foundational support for studies exploring pathological mechanisms. In addition, a smaller proportion of cited journals originates from psychology and nursing.

### 3.6. Highly Co-Cited References

Table 5 presents the top 10 co-cited references that constitute the main intellectual base of PSCI intervention research, rather than a direct representation of the intervention evidence base. These references received 37–112 co-citations and were published between 1975 and 2022. They mainly comprised cognitive assessment tools, epidemiological studies, reviews of pathological mechanisms, the VCI literature, clinical rehabilitation guidelines, and studies on neuromodulation. The citing sources included authoritative journals in neurology, stroke, geriatrics, and general medicine, such as Stroke, Lancet Neurology, BMC Medicine, and European Journal of Neurology. Most highly co-cited items were widely used cognitive scales, systematic reviews, meta-analyses, mechanistic reviews, and clinical guidelines. In particular, publications related to the Montreal Cognitive Assessment (MoCA), Mini-Mental State Examination (MMSE), PSCI epidemiology, and vascular mechanisms had the highest co-citation counts, indicating their strong influence on the conceptual and methodological framework of the field. However, these co-cited references primarily provide assessment tools, disease concepts, and synthesized knowledge for intervention research; they do not directly constitute evidence of therapeutic efficacy. Notably, among the top 10 co-cited references in Table 5, only one was a primary intervention randomized controlled trial (RCT) evaluating treatment efficacy.

Figure S8 displays the top 10 references with citation bursts in PSCI, with bursts spanning 2021–2025. References related to interventions and evidence synthesis continued to show citation bursts through 2025, suggesting sustained academic interest in these topics. However, citation bursts reflect attention and influence within the literature, not the strength of clinical evidence; therefore, these findings can only be used to infer possible research directions, not to establish intervention efficacy.

### 3.7. Keywords

To systematically identify research hotspots in post-stroke cognitive impairment, this study conducted an analysis from three dimensions: keyword co-occurrence, cluster evolution, and keyword burst analysis. The keyword co-occurrence network in Figure 6 shows that “stroke”, “post-stroke cognitive impairment”, and “cognitive function” are the core themes of the field. The keyword clustering analysis identified nine thematic clusters, labeled as vascular dementia, cognitive function, virtual reality, transcranial direct current stimulation, transcranial magnetic stimulation, cognitive impairment, repetitive transcranial magnetic stimulation, cerebral ischemia, and blood pressure. These themes are closely interconnected, with intervention-related keywords exhibiting high co-occurrence intensity with core topics, indicating growing academic attention to intervention-related topics, although this does not by itself establish the clinical effectiveness or maturity of these interventions. The cluster analysis in Figure S9 further suggests an evolution of research themes, with early work centered on pathological and cognitive assessments of vascular dementia and more recent studies increasingly exploring targeted intervention modalities such as non-invasive brain stimulation (NIBS) and virtual reality (VR), including rTMS and tDCS. The keyword burst analysis in Figure S10 demonstrates that burst terms from 2021 to 2022 mainly focused on functional recovery assessment, while the focus shifted to neural mechanism exploration in 2023. Keywords with sustained bursts from 2023 to 2025 include repetitive transcranial magnetic stimulation, tDCS, prefrontal cortex, and outcome assessment, indicating that these topics have recently attracted sustained research attention.

## 4. Discussion

Bibliometric analysis enables a systematic examination of the research landscape, developmental trends, and thematic focus within a field. In this study, CiteSpace and VOSviewer were employed to map PSCI intervention-related publications. We chose 2005–2025 because bibliometric studies typically use a 20-year window. A preliminary WOSCC search found few pre-2005 publications directly related to clinical interventions for PSCI. Including earlier years could reduce the stability and interpretability of network analysis. The MoCA, published in 2005, has since been widely used. Our results show sustained growth over the past two decades and an increasing emphasis on intervention-oriented topics, including neuromodulation approaches such as non-invasive brain stimulation, as reflected by keyword co-occurrence and burst patterns. Compared with earlier bibliometric analyses in related areas such as PSCI or VCI, which have often focused on broad publication trends or non-intervention themes, the present study places a specific emphasis on clinical intervention modalities in PSCI [34,35,36]. By integrating keyword clustering and burst detection with an explicit sub-analysis of primary intervention trials, our work provides a more intervention-centered map of evolving topics and emerging frontiers, thereby offering strategically useful information for methodological standardization and future clinical translation.

### 4.1. Publication Trends

The steady rise in publications investigating PSCI interventions mirrors the advancing maturation of stroke rehabilitation research. Advancements in reperfusion therapies have boosted survival rates among stroke survivors, yet the high prevalence of PSCI has become a prominent clinical challenge, rendering long-term cognitive prognosis a core research priority [24]. Nevertheless, the growth in publication volume does not translate to commensurately robust clinical evidence. A large share of existing intervention trials remains exploratory, limited by small sample cohorts, brief intervention cycles, highly variable participant inclusion thresholds, and inconsistent outcome assessment across studies [37,38,39]. The absence of standardized diagnostic criteria for PSCI further hinders cross-trial comparative analyses [40]. Furthermore, post-stroke cognitive trajectories are dynamically modulated by multiple confounding variables, including infarct location, premorbid cognitive reserve, and cerebral white matter integrity. Such multifactorial influences make it challenging to disentangle the independent therapeutic effects of individual interventions [41,42].

### 4.2. Major Contributors

Research on PSCI interventions presents a dual-core research landscape centered on China and the USA, with the two countries holding distinct yet mutually complementary research advantages. China tops the global ranking in total publication volume; this pattern may reflect its large stroke burden and increasing attention to rehabilitation and cognitive outcomes. A striking characteristic of Chinese scholarship is the high share of studies exploring traditional Chinese medicine (TCM) interventions, such as herbal prescriptions and acupuncture. These therapeutic approaches occupy a vital place in domestic rehabilitation practice and have fostered a relatively mature supporting research system [43,44,45]. Nevertheless, such TCM modalities appear to be less represented in Western academic contexts. By contrast, the USA achieves higher betweenness centrality in global academic collaboration networks and delivers superior citation impact per published article when normalized by time, as indicated by a higher average annual citations per paper than China. This pattern could partly reflect broader international collaboration, higher visibility in English-language journals, and the influence of widely cited clinical statements and guidelines [3,4]. Notably, several countries exhibited strong time normalized citation impact despite lower publication output, particularly Australia (15.5), Germany (12.65), and The Netherlands (10.59) in average annual citations per paper.

At the institutional level, our collaboration and productivity mapping shows that PSCI intervention research is concentrated in a limited number of core centers, suggesting that research capacity and expertise may be clustered. At the thematic level, keyword co-occurrence and burst detection analyses indicate increasing attention to neuromodulation topics, notably rTMS and tDCS, in recent years. However, bibliometric analyses cannot determine protocol-level details such as stimulation targets, stimulation parameters, session schedules, treatment duration, delivery settings, or comparative treatment effect sizes. Evidence from clinical trials and reviews suggests that neuromodulation studies in PSCI use heterogeneous protocols and vary in targets, parameters, dosing schedules, and whether stimulation is combined with cognitive rehabilitation or computerized training [17,46,47,48]. This lack of protocol harmonization limits cross-study comparability and may contribute to fragmentation of intervention themes in the literature. Similar heterogeneity and ongoing debate have also been reported for other intervention modalities, including acupuncture, traditional exercise therapies, virtual reality, and computerized cognitive training [49,50,51].

Author collaboration networks show a fragmented pattern, suggesting that PSCI intervention research capacity is distributed across multiple groups and that large, stable multicenter consortia remain limited. This fragmentation is mirrored by methodological heterogeneity in outcome measurement and follow-up schedules, which complicates cross-study comparability. Moreover, among highly productive authors, time normalized impact varied; for example, Hu, Xiquan (9.52), Luo, Jing (9.47), and De Luca, Rosaria (8.63) showed relatively high average annual citations per paper (Table 3).

Our co-citation analysis indicates that MoCA and MMSE constitute a dominant part of the intellectual base of PSCI intervention-related research. This dominance likely reflects their widespread adoption as pragmatic, easily interpretable screening tools that facilitate comparisons across cohorts, trials, and evidence syntheses, as well as their frequent use as shared endpoints in the literature. At the same time, clinical reports and guideline discussions note that global screening instruments can be affected by stroke-related deficits such as aphasia and motor impairment, and that cutoffs and assessment timing vary across settings [4,52]. Consistent with these concerns, there is increasing use of stroke-adapted or domain-specific tools, including the Oxford Cognitive Screen (OCS) and broader neuropsychological batteries, as well as multidimensional outcomes that extend beyond cognition to mood, activities of daily living (ADL), and, in some studies, imaging markers. The Repeatable Battery for the Assessment of Neuropsychological Status (RBANS) is also frequently reported as a cognitive outcome measure [53].

Importantly, burst detection and temporal trend analyses suggest a recent increase in attention to intervention-oriented topics, particularly neuromodulation approaches such as rTMS and tDCS. From a bibliometric perspective, this pattern is consistent with a gradual shift in research emphasis from assessment and conceptual frameworks toward intervention development and evidence synthesis. However, the co-citation core remains dominated by assessment instruments, reviews, and guidelines, indicating that the field is still in transition and that further methodological standardization is needed. In particular, follow-up time points remain inconsistent across studies, ranging from immediate post-intervention assessments to follow-ups at one, three, or six months, underscoring the need for more harmonized outcome timing and core outcome sets in future trials. Differences in assessment timing may influence the detection rate of cognitive impairment [29,54]. This variability may further limit comparability across existing studies and hinder the development of robust evidence.

The journal distribution also reflects the developmental stage of this field. Research is primarily concentrated in neurology and rehabilitation medicine journals, while extensively citing the basic neuroscience literature, demonstrating a clear translational medicine characteristic. However, representation in top-tier general medical journals remains limited, which may suggest that, despite growing interest, additional confirmatory studies and multicenter trials are needed to strengthen the intervention evidence and improve its visibility and uptake in broad clinical practice.

Overall, although PSCI intervention research is active, it remains structurally decentralized and dispersed. Future progress may benefit from establishing coordinated multicenter research infrastructure, promoting standardized intervention protocols, and strengthening cross-institutional data integration, thereby transforming dispersed research findings into robust and clinically meaningful evidence.

### 4.3. Development History

Our bibliometric analyses depict the evolution of research attention and intellectual structure in the PSCI intervention-related literature. The co-citation patterns of the top 10 influential references (1975–2022) outline the field’s intellectual base. Early highly co-cited studies mainly focused on VCI-related conceptual frameworks and classifications and cerebrovascular mechanisms linked to cognitive impairment. Subsequent influential references reflect increasing use of the PSCI construct to describe cognitive decline temporally associated with stroke [27]. Throughout this period, cognitive assessment instruments have exerted the most enduring citation influence. The widespread adoption of the MoCA and MMSE stems not only from their robust psychometric properties but also from their capacity to address the historical lack of standardized diagnostic metrics [27]. Nevertheless, the predominance of these screening tools underscores a historical research emphasis on phenotypic identification and outcome quantification, rather than on mechanism-guided therapeutic development. Co-cited rehabilitation guidelines reflect efforts toward clinical translation and frequently note limitations in the available intervention evidence [30,33]. Notably, primary intervention RCTs were less represented among the most influential co-cited references. Among the top co-cited references, only one rTMS trial appeared, which reflects the co-citation structure and may be influenced by citation lag and database coverage rather than the overall availability of efficacy trials [32]. Recent burst keywords and burst references related to intervention modalities and evidence synthesis indicate increasing scholarly attention to these topics.

### 4.4. Current Research Hotspots and Future Directions

Keyword analysis shows that PSCI intervention research is moving from a focus on disease identification and functional assessment toward mechanism-driven and intervention optimization models. Three major research hotspots have emerged: NIBS, computerized cognitive training, and VR. Importantly, although keyword co-occurrence, cluster evolution, and citation bursts reveal that NIBS, VR, and mechanism-related studies have become increasingly prominent in PSCI research, these findings should be interpreted as indicators of research popularity and evolving thematic focus, not as evidence of clinical effectiveness or maturity. The transition toward “precision rehabilitation” suggested by these bibliometric patterns reflects a growing interest in personalized and mechanism-informed approaches, but the actual therapeutic value and clinical readiness of these interventions require validation through well-designed clinical trials and systematic evidence synthesis.

The rise in NIBS reflects interdisciplinary advances in neuroscience and rehabilitation. Repetitive transcranial magnetic stimulation and transcranial direct current stimulation are the primary research modalities. rTMS achieves higher spatial resolution and deeper cortical penetration, while tDCS is widely adopted for home rehabilitation due to its safety, portability and lower cost [55]. Existing evidence indicates that combining stimulation with task-specific training may generate synergistic effects. Computerized cognitive training paired with tDCS can further improve cognition and ADL in PSCI patients, particularly visuospatial and executive function [55]. Intermittent theta-burst stimulation (iTBS) combined with cognitive training improves cognitive function, upper-limb movement and gait performance, with cognitive scores significantly correlated with spatiotemporal and kinematic gait parameters [56]. Computerized cognitive training possesses unique merits relative to traditional rehabilitation. Patients can receive repeated self-directed training, acquire immediate performance feedback, and adjust task difficulty according to their functional status [21,57]. Studies on brain-injured patients confirm that computerized cognitive training effectively relieves memory, attention and visuospatial deficits, supporting its application in PSCI treatment [58]. VR can generate realistic three-dimensional environments and task scenarios to strengthen interaction between patients and virtual contexts. VR training provides high-intensity, repetitive, multi-sensory and goal-oriented tasks, and training intensity and repetition are critical contributors to neuroplasticity after brain injury [59,60]. TCM interventions are also an important component of PSCI research. Clinical acupuncture lacks unified standardized operating protocols; mechanistic investigations of herbal formulations remain insufficient; and large-scale international replicative trials are rarely conducted. Given the limited evidence supporting pharmacological interventions for PSCI, an ongoing double-blind RCT (ITMCTR2026002185) is evaluating the efficacy and safety of Compound Congrong Yizhi Capsule in patients with PSCI. Its findings are expected to provide high-quality clinical evidence and help address the current evidence gap in the pharmacological management of PSCI.

Overall, PSCI interventions include multiple therapeutic approaches. Each approach shows specific clinical value, but stronger methodological standardization and high-quality comparative trials are needed to strengthen the evidence base.

## 5. Limitation

This study has several limitations. First, all data were retrieved solely from the Web of Science Core Collection. Despite a comprehensive search strategy with multiple keyword combinations, some relevant studies may still have been omitted. Second, the inclusion of only English-language articles and reviews may have introduced selection bias. Third, the database choice and language restriction could systematically affect the observed rankings of countries, institutions, and research topics. Although Chinese institutions contributed the largest number of publications (n = 358), this count likely underestimates China’s total research output because a considerable number of PSCI intervention studies–especially those on traditional Chinese medicine, acupuncture, and herbal formulas—are published in Chinese-language journals indexed in China National Knowledge Infrastructure (CNKI) or the Wanfang database. Conversely, the citation impact of Chinese institutions may also be underestimated, as WoSCC citations primarily reflect international visibility, whereas TCM-related articles tend to receive more citations within Chinese databases. Regarding intervention types, the prominence of TCM interventions is likely underrepresented due to the exclusion of the Chinese-language literature, while emerging interventions such as NIBS, computerized cognitive training, and VR, which are mostly reported in international English-language journals, are probably better captured. Bibliometric results may be influenced by citation-age bias and citation lag, metadata indexing inaccuracies, and imperfect author and institution disambiguation, and no formal study-quality appraisal was performed; therefore, findings reflect publication or citation activity rather than treatment effect sizes. Therefore, citation-based comparisons may be affected by citation-age bias and differences in citation practices across publication years and research areas. Finally, this analysis covers publications up to 31 December 2025, and therefore does not include studies published thereafter. To mitigate the effect of this time lag, we performed keyword burst detection and temporal trend analyses to better identify emerging research directions.

## 6. Conclusions

This bibliometric analysis maps the publication growth and knowledge structure of PSCI intervention-related research over the past two decades, showing sustained expansion with an acceleration after 2015. Based on keyword co-occurrence patterns and burst detection, the literature has increasingly emphasized cognitive assessment frameworks and evidence synthesis, alongside growing attention to mechanistic themes and intervention-oriented topics such as neuromodulation and rehabilitation strategies. Citation bursts also suggest rising interest in outcome measurement and reporting consistency, highlighting the need for more standardized assessment and comparability across studies. Future advances may be facilitated by stronger international collaboration to harmonize study designs, outcome measures, and reporting standards, thereby improving cross-site comparability and supporting the translation of promising interventions into robust, generalizable clinical evidence.

## Figures and Tables

**Figure 1:**
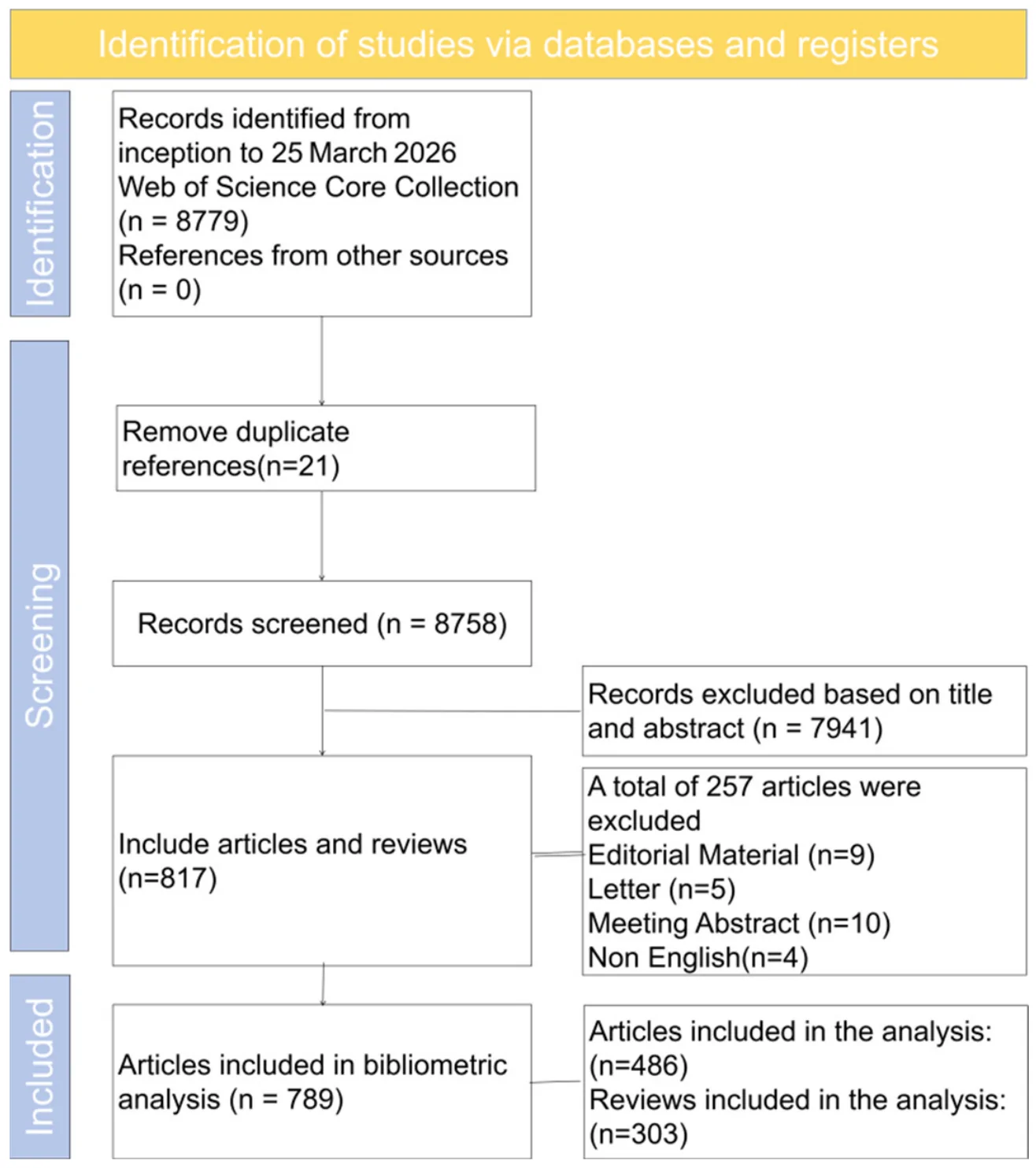
Flowchart of the bibliometric analysis procedure for post-stroke cognitive impairment (PSCI) intervention research. All the eligible literature was retrieved from the Web of Science Core Collection (WoSCC). After strict screening, valid publications were imported for visual analysis. Bibliometric networks and trend analyses were systematically performed using VOSviewer and CiteSpace software to clarify the research status, hotspots, and development trends of PSCI intervention studies.

**Figure 2:**
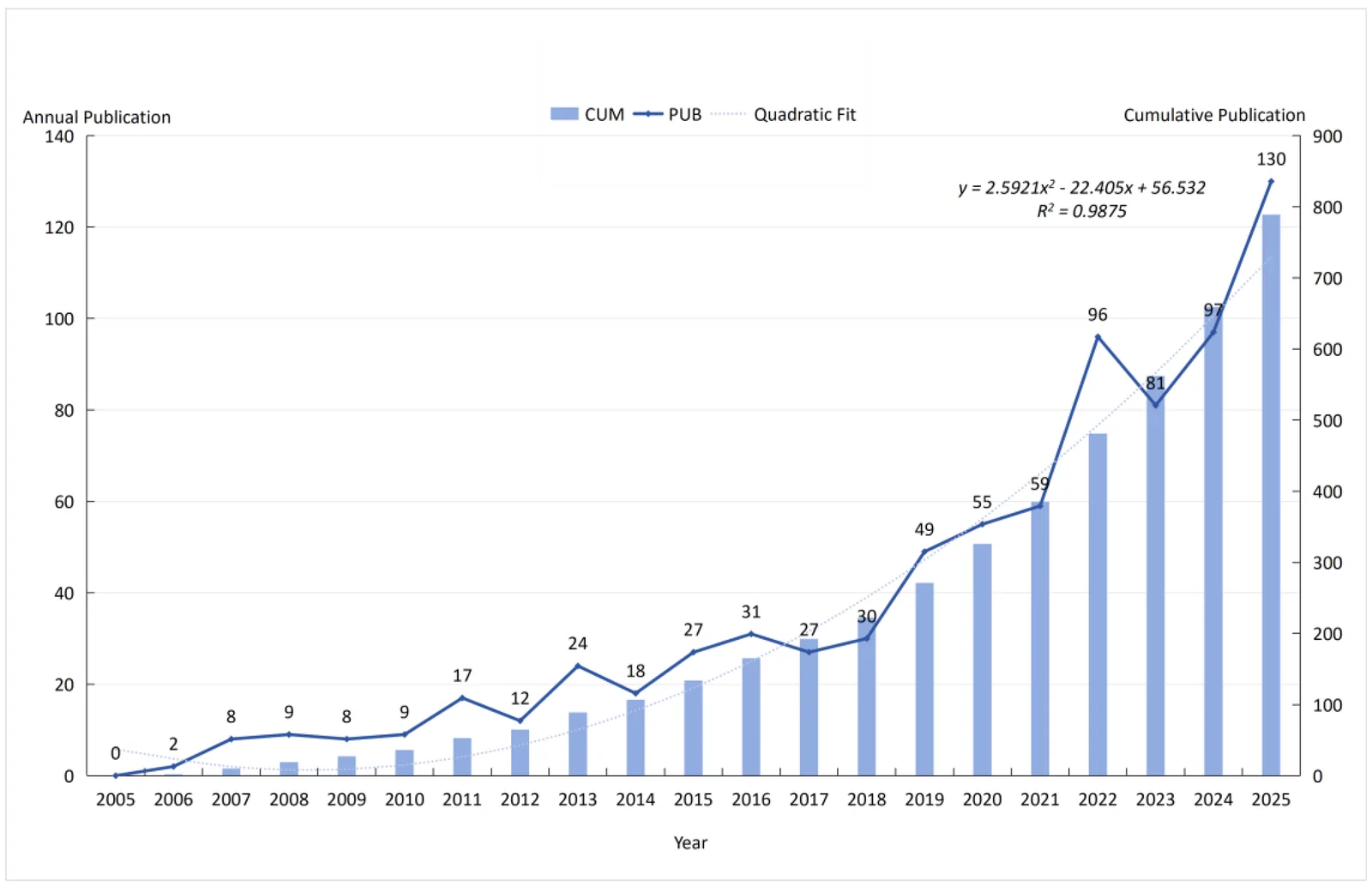
Annual and cumulative publication trends of studies on post-stroke cognitive impairment (PSCI) interventions from 2005 to 2025.

**Figure 3:**
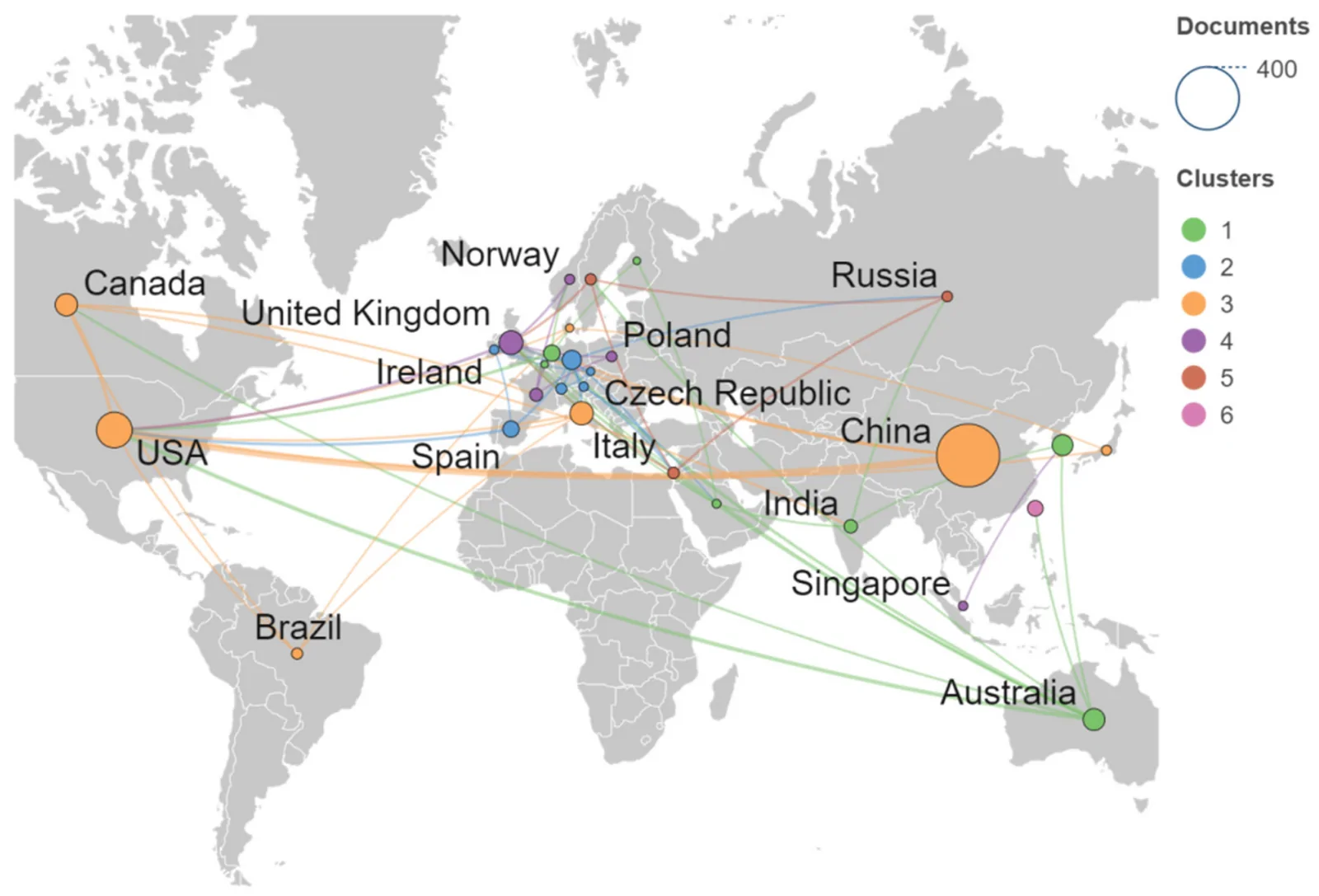
Global country/region collaboration network in post-stroke cognitive impairment (PSCI) intervention research. Nodes represent countries/regions, and node size is proportional to the number of publications. Links indicate international co-authorship relationships, with thicker links representing stronger collaboration.

**Figure 4:**
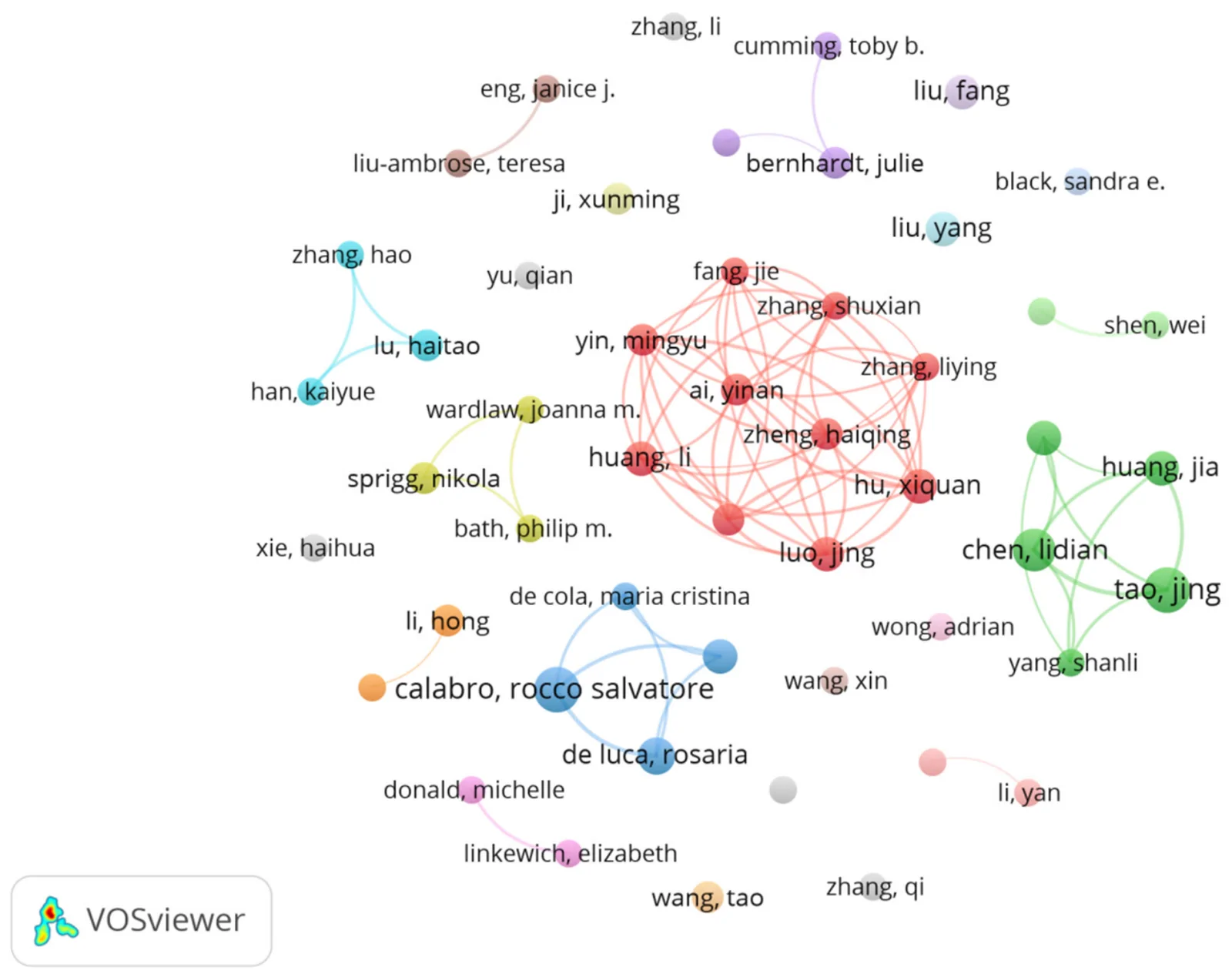
Co-authorship network of authors in post-stroke cognitive impairment (PSCI) intervention research. Nodes represent authors, and node size is proportional to the number of publications. Links indicate co-authorship relationships, with thicker links reflecting stronger collaboration. Colors denote clusters of authors who collaborate more closely with each other.

**Figure 5:**
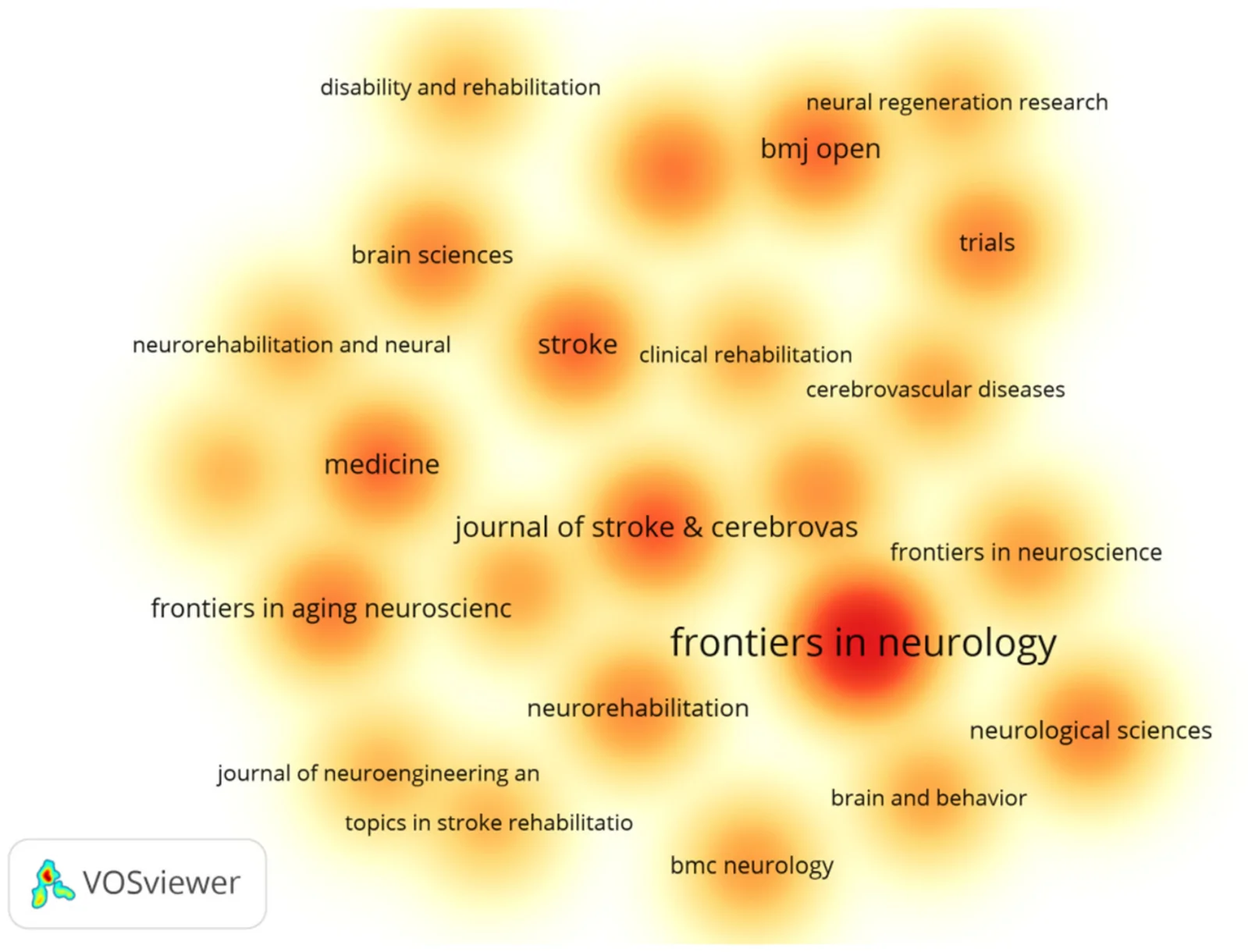
Density visualization of source journals publishing post-stroke cognitive impairment (PSCI) intervention research. Each label represents a journal. The color gradient from light yellow to red indicates increasing publication density (higher concentration of articles), with red areas denoting journals that contribute more publications and occupy more central positions in the journal distribution of this field.

**Figure 6:**
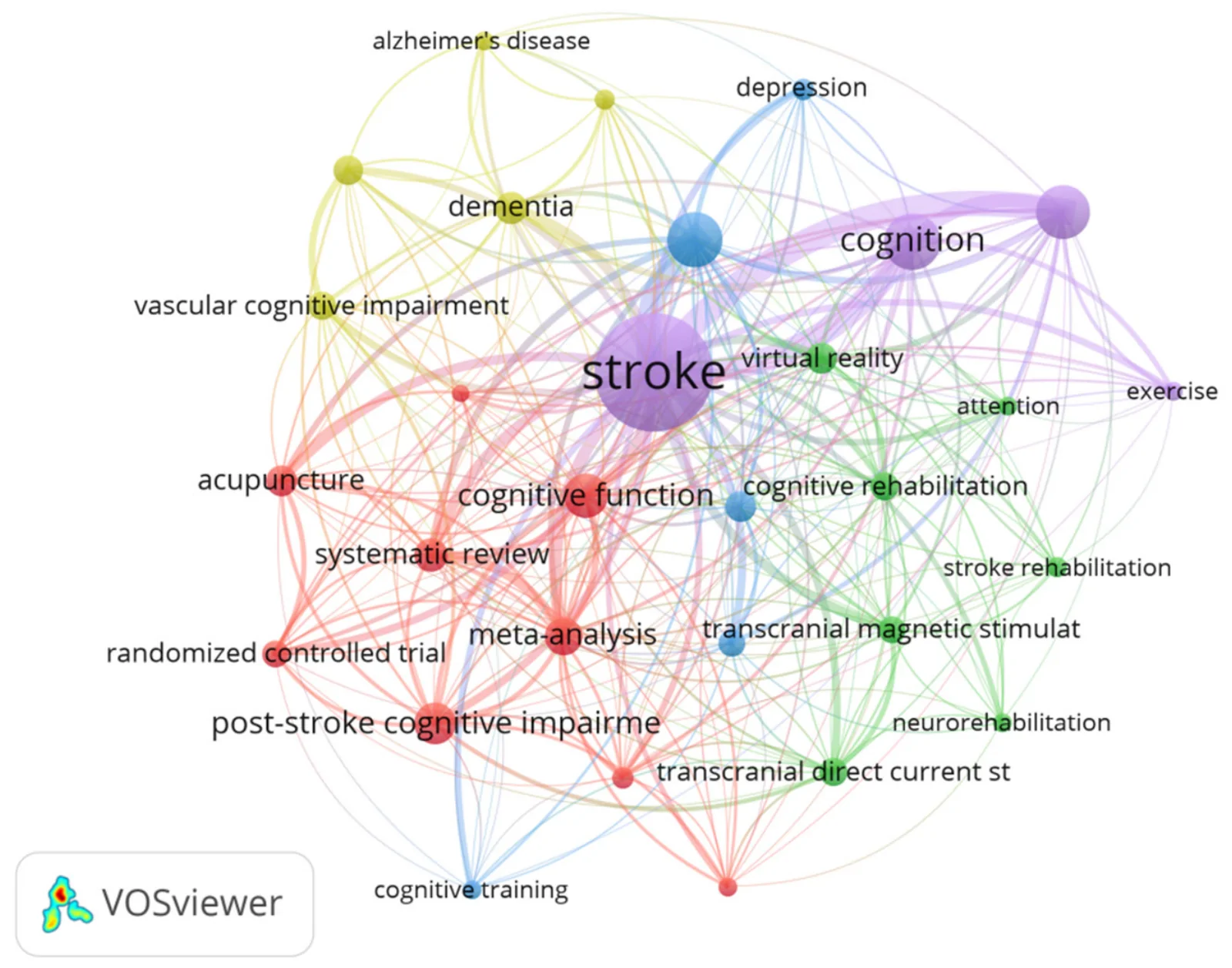
Keyword co-occurrence network of post-stroke cognitive impairment (PSCI) intervention research. Nodes represent keywords, with node size proportional to occurrence frequency. Links indicate co-occurrence relationships between keywords, and thicker links represent stronger co-occurrence. Different colors denote keyword clusters, reflecting major thematic groups in the field.

**Table 1:** Top 10 countries/regions by publication count.

Rank	Country/Region	Publications	Citations	Average Annual Citations per Paper	Centrality
1	China	358	6924	5.19	0.17
2	USA	115	6490	8.62	0.48
3	United Kingdom	49	2974	6.94	0.03
4	Italy	47	2435	7.32	0.21
5	Canada	45	3093	7.86	0.09
6	Australia	43	3822	15.5	0.16
7	South Korea	39	1214	5.33	0.09
8	Germany	33	2741	12.65	0.12
9	The Netherlands	24	1717	10.59	0.06
10	Spain	24	594	4.03	0.04

Documents denotes the total number of papers published. Citations represent the cumulative number of citations received. Average Annual Citations per Paper measures the mean yearly citation impact per paper. Centrality reflects a country/region’s bridging (mediating) role in the international cooperation network; higher values indicate stronger intermediary influence.

**Table 2:** Top 10 institutions by publication count.

Rank	Institution	Documents	Citations	Average Citations	Average Annual Citations per Paper	Country
1	Capital Medical University	25	391	15.6	4.63	China
2	Fujian University of Traditional Chinese Medicine	22	681	31.0	5.7	China
3	University of Toronto	16	1542	96.4	9.91	Canada
4	Monash University	15	1550	103.3	22.05	Australia
5	Shanghai University of Traditional Chinese Medicine	14	212	15.1	3.69	China
6	Tianjin University of Traditional Chinese Medicine	13	232	17.8	5.04	China
7	University of Nottingham	12	926	77.1	9.56	United Kingdom
8	Zhejiang University	12	235	19.6	4.12	China
9	Sun Yat-sen University	12	307	25.6	6.31	China
10	The Chinese University of Hong Kong	12	444	37.0	5.7	China

Documents denotes the total number of published papers. Citations represents the cumulative number of citations received. Average citations indicates the mean citations per paper. Average annual citations per paper refers to the average number of citations received per paper per year. Country indicates the country/region of the institution.

**Table 3:** The 12 authors with ≥6 publications.

Rank	Author	Documents	Citations	Average Citations	Average Annual Citations per Paper	Country
1	Calabro, Rocco Salvatore	10	165	16.5	7.21	Italy
	Tao, Jing	10	311	31.1	3.51	China
2	Chen, Lidian	9	340	37.8	4.83	China
3	De Luca, Rosaria	7	383	54.7	8.63	Italy
4	Hu, Xiquan	6	311	51.8	9.52	China
5	Huang, Jia	6	358	59.7	2.85	China
6	Huang, Li	6	213	35.5	6.18	China
7	Luo, Jing	6	113	18.8	9.47	China
8	Maggio, Maria Grazia	6	137	22.8	6.71	Italy
10	Zheng, Guohua	6	57	9.5	5.09	China
11	Liu, Fang	6	198	33.0	8.28	China
12	Liu, Yang	6	64	7.1	4.48	China

Documents denotes the total number of published papers. Citations represents the cumulative number of citations received. Average citations indicates the mean citation count per paper. Average annual citations per paper refers to the average number of citations received per paper per year. Country indicates the author’s country/region.

**Table 4:** The 10 journals by publication counts.

Rank	Journal	Documents	Citations	Average Annual Citations per Paper	IF (JCR2024)	JCR Quartile	Country
1	Frontiers in Neurology	48	728	4.2	2.8	Q2	Switzerland
2	Journal of Stroke & Cerebrovascular Diseases	21	809	5.37	1.8	Q3	USA
3	BMJ Open	18	205	2.02	2.3	Q2	United Kingdom
4	Medicine	18	147	2.05	1.4	Q2	USA
5	Stroke	18	1668	12.85	8.9	Q1	USA
6	Cochrane Database of Systematic Reviews	16	2094	17.84	9.4	Q1	United Kingdom
7	Frontiers in Aging Neuroscience	15	294	5.64	4.5	Q1	Switzerland
8	Brain Sciences	14	189	4.58	2.8	Q3	Switzerland
9	Neurological Sciences	14	182	6.05	2.4	Q3	Italy
10	Trials	14	95	1.13	2.0	Q3	United Kingdom

Documents denotes the total number of published papers. Citations represents the cumulative number of citations received. Average annual citations per paper refers to the average number of citations received per paper per year. IF (JCR2024) represents the journal impact factor (IF) reported in the 2024 Journal Citation Reports (JCR). JCR quartile indicates the journal’s quartile ranking (Q1–Q4) within its subject category in JCR.

**Table 5:** The top 10 co-cited references.

Rank	Title	Year	Journal	First Author	Co-Citations	DOI
1	The montreal cognitive assessment, MoCA: A brief screening tool for mild cognitive impairment [25]	2005	Journal of the American Geriatrics Society	Nasreddine, ZS	112	10.1111/j.1532-5415.2005.53221.x
2	Mini-mental state. A practical method for grading the cognitive state of patients for the clinician [26]	1975	Journal of Psychiatric Research	Folstein, MF	69	10.1016/0022-3956(75)90026-6
3	Prevalence, incidence, and factors associated with pre-stroke and post-stroke dementia: a systematic review and meta-analysis [27]	2009	Lancet Neurology	Pendlebury, ST	67	10.1016/S1474-4422(09)70236-4
4	Post-stroke cognitive impairment: epidemiology, mechanisms and management [28]	2014	Annals of Translations Medicine	Sun, JH	65	10.3978/j.issn.2305-5839.2014.08.05
5	Post-Stroke Cognitive Impairment and Dementia [29]	2022	Circulation Research	Rost, S R	57	10.1161/CIRCRESAHA.122.319951
6	Vascular Contributions to Cognitive Impairment and Dementia A Statement for Healthcare Professionals From the American Heart Association/American Stroke Association [30]	2011	Stroke	Gorelick, PB	54	10.1161/str.0b013e3182299496
7	Post-stroke dementia—a comprehensive review [5]	2017	BMC Medicine	Mijajlovic, MD	52	10.1186/s12916-017-0779-7
8	Post-stroke cognitive impairment is common even after successful clinical recovery [31]	2015	European Journal of Neurology	Jokinen, H	48	10.1111/ene.12743
9	Effects of rTMS Treatment on Cognitive Impairment and Resting-State Brain Activity in Stroke Patients: A Randomized Clinical Trial [32]	2020	Frontiers in Neural Circuits	Yin, MY	40	10.3389/fncir.2020.563777
10	Guidelines for Adult Stroke Rehabilitation and Recovery A Guideline for Healthcare Professionals From the American Heart Association/American Stroke Association [33]	2016	Stroke	Winstein, CJ	37	10.1161/str.0000000000000098

## Data Availability

No new data were created or analyzed in this study. Data sharing is not applicable to this article.

## Supplementary Materials

The following supporting information can be downloaded at: https://www.mdpi.com/article/10.3390/healthcare14193210/s1, Figure S1: Chord diagram of international collaboration in post-stroke cognitive impairment (PSCI) intervention research; Figure S2: Density heatmap of country/region contributions to PSCI intervention research. Each label represents a country/region; Figure S3: Inter-institutional collaboration network for PSCI intervention research; Figure S4: Institutional collaboration network of the top 10 productive institutions in PSCI intervention research; Figure S5: Co-authorship network of productive authors in PSCI intervention research; Figure S6: Journal co-citation network of PSCI intervention research; Figure S7: Cross-disciplinary distribution map of Web of Science categories; Figure S8: Top 10 references with the strongest citation bursts; Figure S9: Keyword clustering map drawn by CiteSpace; Figure S10: Top 10 keywords with the strongest citation bursts; Table S1: The search strategy; Table S2: Keyword cluster robustness metrics.

## Abbreviations

ADL	activities of daily living
CNKI	China National Knowledge Infrastructure
IF	journal impact factor
iTBS	intermittent theta-burst stimulation
JCR	journal citation reports
MoCA	Montreal Cognitive Assessment
MMSE	Mini-Mental State Examination
NIBS	non-invasive brain stimulation
PSCI	post-stroke cognitive impairment
PSD	post-stroke dementia
RCT	randomized controlled trial
RBANS	repeatable battery for the assessment of neuropsychological status
rTMS	repetitive transcranial magnetic stimulation
tDCS	transcranial direct current stimulation
TCM	traditional Chinese medicine
VCI	vascular cognitive impairment
VR	virtual reality
WoSCC	Web of Science Core Collection
