# Supplementary material for: Global Trends and Research Hotspots of Interventions for Post-Stroke Cognitive Impairment: A Bibliometric Analysis from 2005 to 2025
Source: Healthcare (Basel). 2026 Sep 28;14(19):3210. doi: 10.3390/healthcare14193210 (PMC13665520; doi:10.3390/healthcare14193210)
Supplement: Supplementary file 1 [file healthcare-14-03210-s001.zip › healthcare-4524465-supplementary.pdf]

S1 Supplementary Table

Table S1 The search strategy

The search strategy for Web of Science core collection	
Number	Search terms
#1	TS=("post-stroke cognitive impairment" OR "post-stroke cognitive deficits" OR "post-stroke cognitive disorder" OR "cognitive impairment after stroke" OR "post-stroke dementia" OR "vascular cognitive impairment no dementia after stroke")
#2	Stroke[MeSH Terms] were searched in Pubmed
	Ischaemic Stroke[MeSH Terms] were searched in Pubmed
	Cerebral Hemporrhage[MeSH Terms] were searched in Pubmed
	TS=(stroke* OR "cerebrovascular accident*" OR "cerebral stroke*" OR "cerebrovascular apoplexy*" OR apoplexy* OR CVA OR "acute stroke*" OR "acute cerebrovascular accident*" OR "brain vascular accident*" OR "Isch?emic Stroke*" OR "cryptogenic isch?emic stroke*" OR "cryptogenic embolic stroke*" OR "cryptogenic stroke*" OR "wake-up stroke*" OR "wake up stroke*" OR "cerebral hemorrhage*" OR "cerebral haemorrhage*" OR "intracerebral hemorrhage*" OR "intracerebral haemorrhage*" OR "cerebrum hemorrhage*" OR "cerebrum haemorrhage*" OR "cerebral parenchymal hemorrhage*" OR "cerebral parenchymal haemorrhage*")
#3	Cognitive Dysfunction[MeSH Terms] were searched in Pubmed
	TS=("cognitive dysfunction*" OR "cognitive disorder*" OR "cognitive impairment*" OR "mild cognitive impairment*" OR "cognitive decline*" OR "mental deterioration*")
#4	TS=(treatment OR therapy OR intervention OR management OR "pharmacological treatment" OR medication OR "drug therapy" OR pharmacotherapy OR "cholinesterase inhibitor*" OR "acetylcholinesterase

	inhibitor*" OR "NMDA receptor antagonist*" OR donepezil OR galantamine OR rivastigmine OR memantine OR nimodipine OR citicoline OR "CDP-choline" OR "ginkgo biloba" OR EGb761 OR cerebrolysin OR acupuncture OR "herbal medicine" OR "TCM treatment" OR "traditional Chinese medicine" OR "non-pharmacological intervention" OR rehabilitation OR "cognitive training" OR "cognitive rehabilitation" OR neuromodulation OR "brain stimulation" OR "non-invasive brain stimulation" OR NIBS OR "transcranial magnetic stimulation" OR TMS OR "repetitive transcranial magnetic stimulation" OR rTMS OR "theta burst stimulation" OR TBS OR "continuous theta burst stimulation" OR cTBS OR "intermittent theta burst stimulation" OR iTBS OR "transcranial direct current stimulation" OR tDCS OR "transcranial alternating current stimulation" OR tACS OR "transcranial random noise stimulation" OR tRNS)
#5	#2 AND# 3
#6	#1 OR# 5
#7	#6 AND# 4

Table S2 Keyword cluster robustness metrics

Cluster ID	Label	Size (n keywords)	Silhouette	Mean year
0	vascular dementia	92	0.664	2012
1	cognitive function	66	0.715	2016
2	virtual reality	62	0.639	2016
3	transcranial direct current stimulation	62	0.718	2016
4	transcranial magnetic stimulation	55	0.670	2018
5	cognitive impairment	40	0.784	2011
6	repetitive transcranial magnetic	38	0.741	2019

	stimulation			
7	cerebral ischemia	17	0.894	2012
8	blood pressure	12	0.983	2009

Overall modularity Q = 0.3704; weighted mean silhouette S = 0.7111.

S2 Supplementary Figure

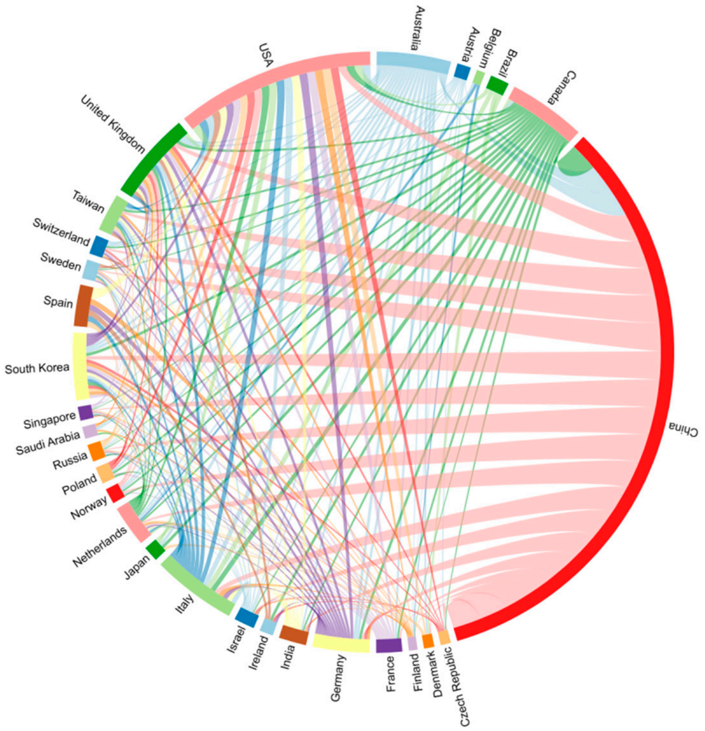

Figure S1. Chord diagram of international collaboration in post-stroke cognitive impairment （PSCI）intervention research. Each arc on the outer ring represents a country/region, and arc length is proportional to its publication output. Chords connecting two arcs indicate co-authorship collaborations between countries/regions, with chord thickness reflecting collaboration strength.

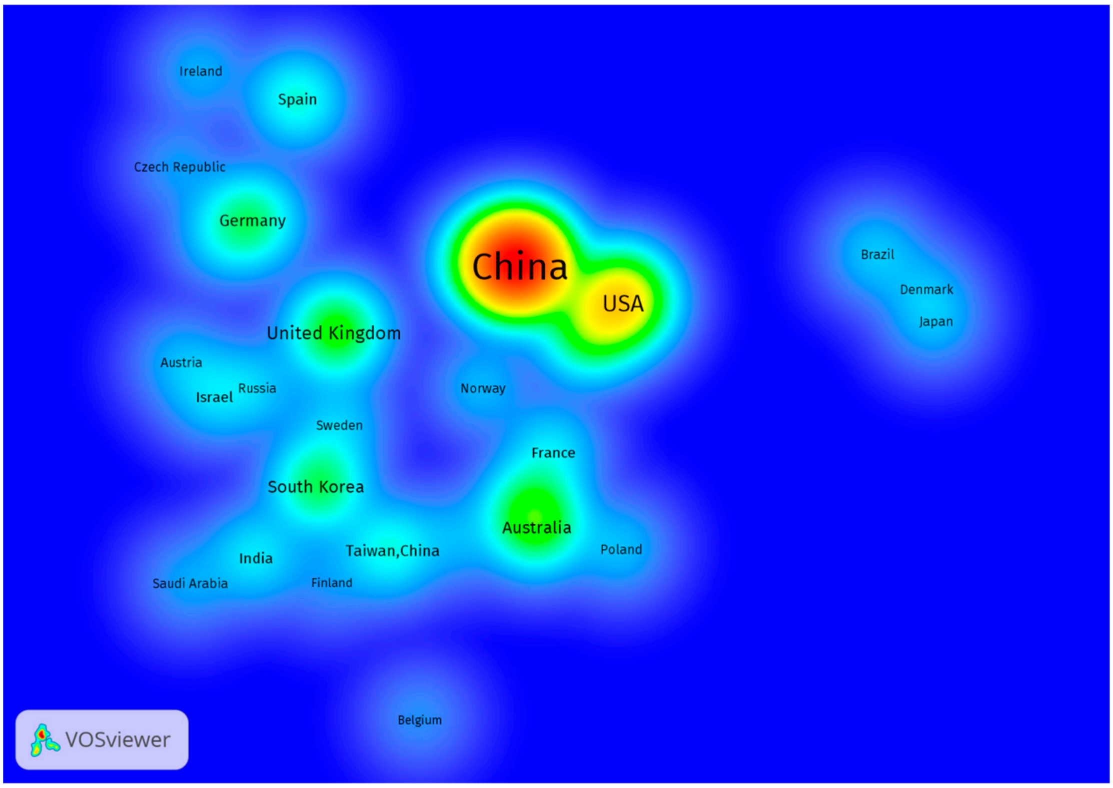

Figure S2. Density heatmap of country/region contributions to post-stroke cognitive impairment （PSCI） intervention research. Each label represents a country/region. The color gradient from blue to red indicates increasing publication density, with warmer colors (yellow-red) denoting higher concentrations of publications and greater research activity.

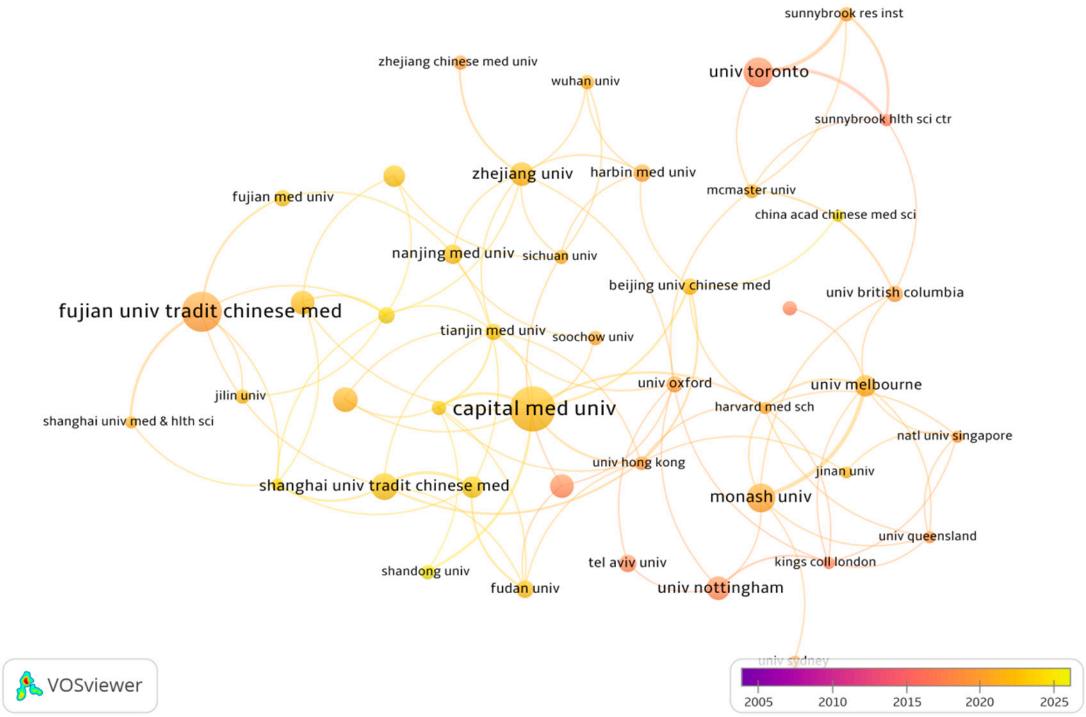

Figure S3. Inter-institutional collaboration network for post-stroke cognitive impairment （PSCI） intervention research. Nodes represent institutions, and node size is proportional to the number of publications. Links indicate co-authorship collaborations between institutions. Node color reflects the average publication year (purple indicates earlier contributions and yellow indicates more recent contributions), illustrating the temporal evolution of institutional participation in this field.

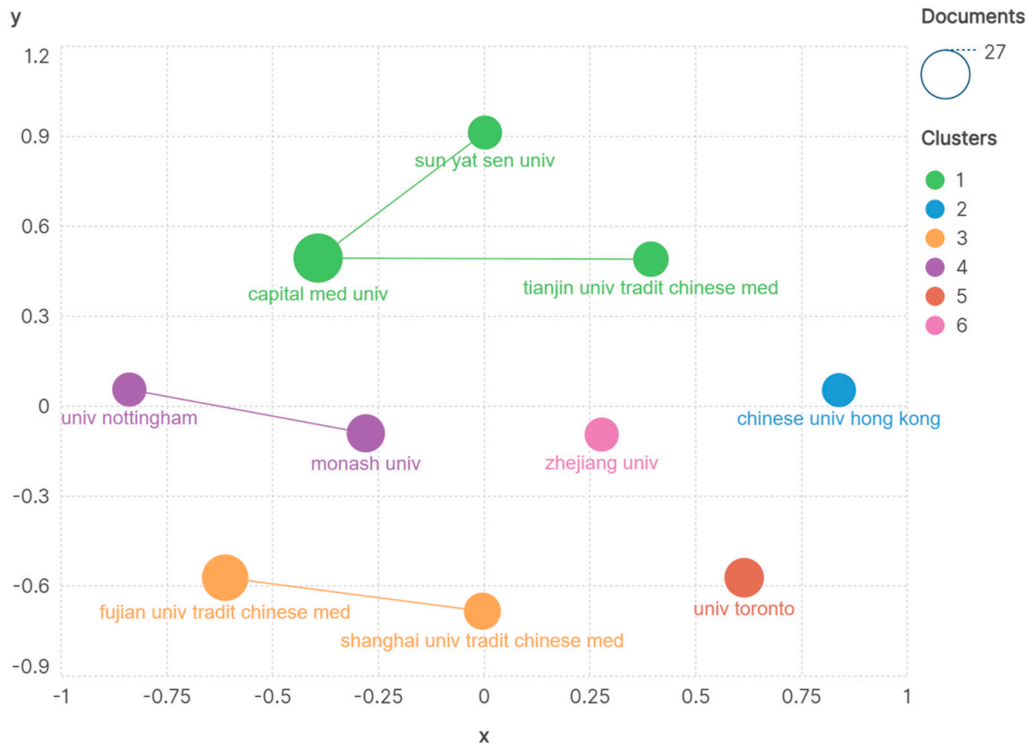

Figure S4. Institutional collaboration network of the top 10 productive institutions in post-stroke cognitive impairment （PSCI） intervention research. Nodes represent institutions, with node size proportional to the number of documents. Links indicate inter-institutional co-authorship relationships. The spatial positions (x-y coordinates) reflect the relative relatedness of institutions (shorter distances indicate stronger association).

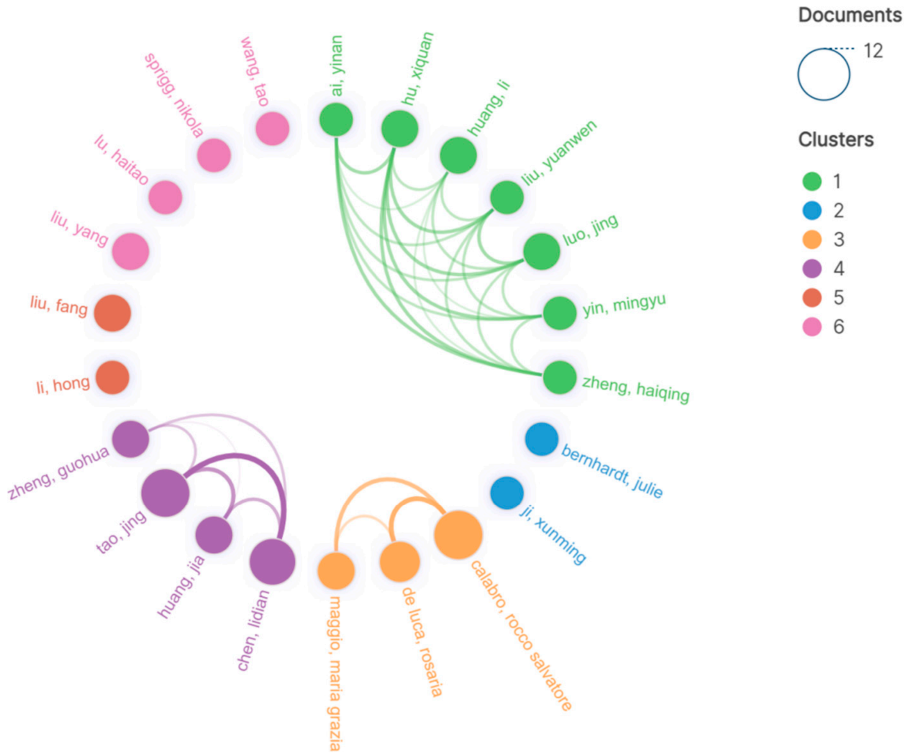

Figure S5. Co-authorship network of productive authors in post-stroke cognitive impairment （PSCI） intervention research. Nodes represent authors and node size is proportional to the number of documents. Curved links indicate co-authorship relationships, with thicker links representing stronger collaboration.

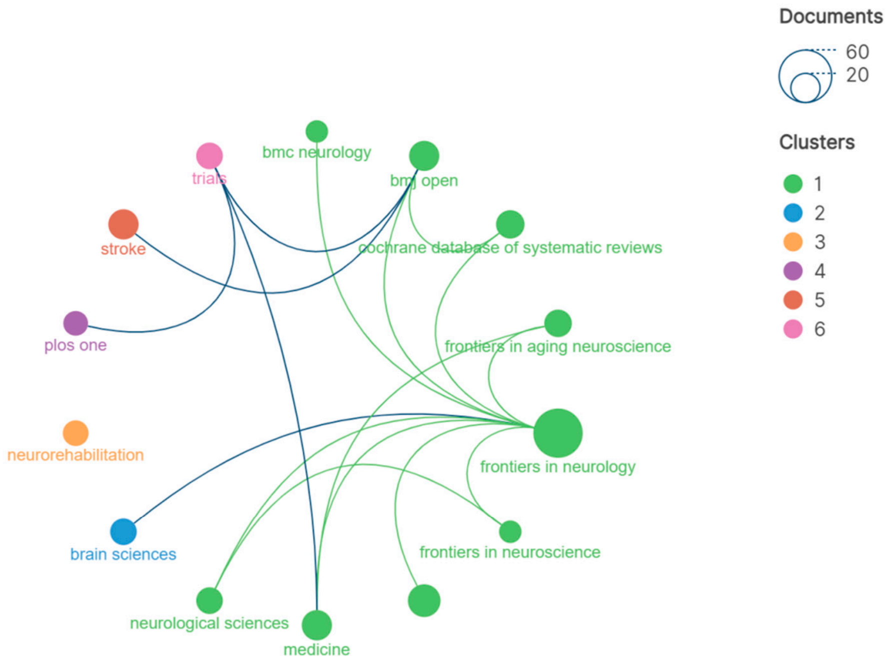

Figure S6. Journal co-citation network of post-stroke cognitive impairment （PSCI） intervention research. Nodes represent journals, with node size proportional to the number of documents. Links indicate co-citation relationships between journals, and thicker links represent stronger co-citation strength.

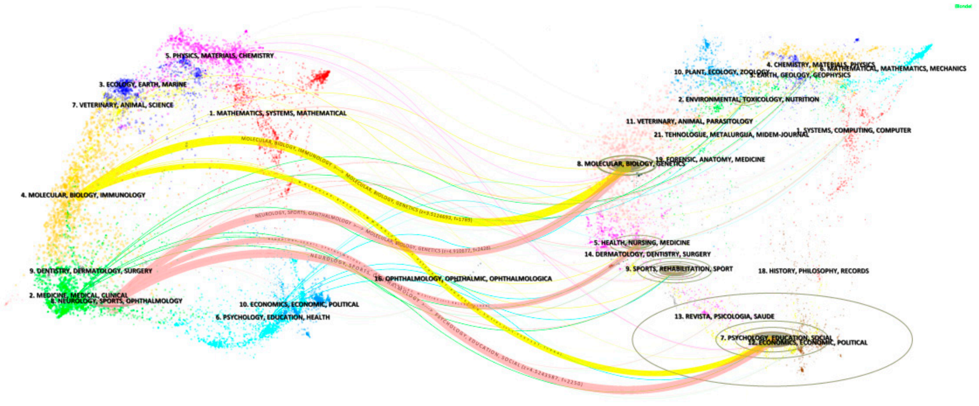

Figure S7. Cross-disciplinary distribution map of Web of Science categories. Color belts show citation links between different disciplines.

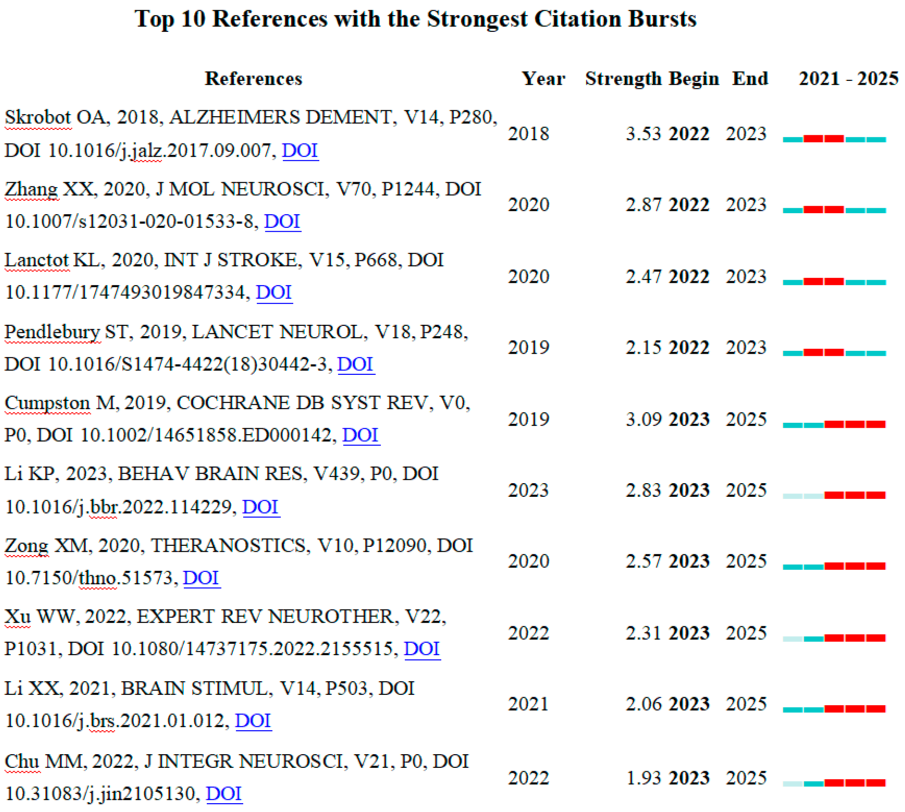

Top 10 References with the Strongest Citation Bursts

References	Year	Strength	Begin	End	2021 - 2025
Skrobot OA, 2018, ALZHEIMERS DEMENT, V14, P280, DOI 10.1016/j.jalz.2017.09.007, DOI	2018	3.53	**2022**	2023	
Zhang XX, 2020, J MOL NEUROSCI, V70, P1244, DOI 10.1007/s12031-020-01533-8, DOI	2020	2.87	**2022**	2023	
Lanctot KL, 2020, INT J STROKE, V15, P668, DOI 10.1177/1747493019847334, DOI	2020	2.47	**2022**	2023	
Pendlebury ST, 2019, LANCET NEUROL, V18, P248, DOI 10.1016/S1474-4422(18)30442-3, DOI	2019	2.15	**2022**	2023	
Cumpston M, 2019, COCHRANE DB SYST REV, V0, P0, DOI 10.1002/14651858.ED000142, DOI	2019	3.09	**2023**	2025	
Li KP, 2023, BEHAV BRAIN RES, V439, P0, DOI 10.1016/j.bbr.2022.114229, DOI	2023	2.83	**2023**	2025	
Zong XM, 2020, THERANOSTICS, V10, P12090, DOI 10.7150/thno.51573, DOI	2020	2.57	**2023**	2025	
Xu WW, 2022, EXPERT REV NEUROTHER, V22, P1031, DOI 10.1080/14737175.2022.2155515, DOI	2022	2.31	**2023**	2025	
Li XX, 2021, BRAIN STIMUL, V14, P503, DOI 10.1016/j.brs.2021.01.012, DOI	2021	2.06	**2023**	2025	
Chu MM, 2022, J INTEGR NEUROSCI, V21, P0, DOI 10.31083/j.jin2105130, DOI	2022	1.93	**2023**	2025	

Figure S8. Top 10 references with the strongest citation bursts. The table records burst strength and burst time. Red bars mark active burst periods.

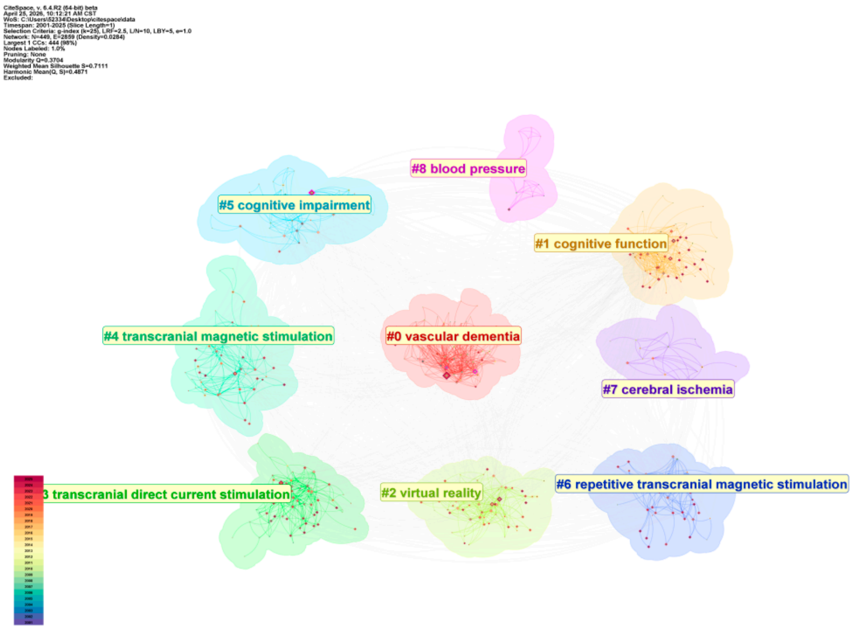

Figure S9. Keyword clustering map drawn by CiteSpace. Each colored area is one research cluster. Color gradient reflects the time sequence of research hostpots.

Top 10 Keywords with the Strongest Citation Bursts

Keywords	Year	Strength	Begin	End	2021 - 2025
performance	2021	2.44	**2021**	2022	
balance	2021	2.14	**2021**	2021	
gait	2021	2.01	**2021**	2021	
networks	2023	2.11	**2023**	2023	
Outcome	2021	1.88	**2023**	2025	
prefrontal cortex	2024	2.2	**2024**	2025	
repetitive transcranial magnetic stimulation	2022	1.87	**2024**	2025	
motor recovery	2022	1.86	**2024**	2025	
transcranial direct current stimulation	2024	1.86	**2024**	2025	
intermittent theta burst stimulation	2024	1.86	**2024**	2025	

Figure S10. Top 10 keywords with the strongest citation bursts. The table lists burst strength and burst duration. Bars show active periods from 2021 to 2025.

S3 Supplementary Method

Supplementary Method S1

To evaluate the temporal impact of the included publications, a time-normalization procedure was applied to calculate the annual citations. First, the publication age for each paper was calculated as the current year minus the publication year plus one, with the publication year counted as year one to avoid a zero denominator. The single-paper annual citations were then determined by dividing the total times cited by the publication age. Subsequently, these single-paper annual citations were aggregated across four dimensions: countries, institutions, authors, and journals. For countries and institutions, a deduplication logic was applied, meaning that if a paper had multiple authors from the same country or institution, it contributed only once to that entity; in cases of international or inter-institutional collaboration, the paper's full annual citation value was credited to each participating entity. For authors, a full counting method was applied, where the paper's full annual citation value was credited to all co-authors. For journals, each paper was assigned to exactly one journal, requiring no deduplication. Finally, three core indicators were calculated for each entity: (1) Publications, the total number of papers attributed to the entity; (2) Total Annual Citations, the sum of the single-paper annual citations for all attributed papers; and (3) Average Annual Citations per Paper, the arithmetic mean of the single-paper annual citations. This final metric eliminates the influence of publication volume, thereby reflecting the average annual impact quality of the entity.
